# A Crumpet, a Canine and a Cryoprobe: A Case of Tooth Aspiration

**DOI:** 10.1002/rcr2.70295

**Published:** 2025-07-24

**Authors:** Matthew Donnan, Melanie Wong, Elina Chi, Dominic Keating

**Affiliations:** ^1^ Department of Respiratory Medicine The Alfred Hospital Melbourne Victoria Australia; ^2^ Faculty of Medicine Nursing and Health Sciences Central Clinical School, Monash University Melbourne Victoria Australia

**Keywords:** aspiration, bronchoscopy, cryoprobe, foreign body, tooth

## Abstract

Foreign body inhalation can lead to post‐obstructive pneumonia and sepsis, requiring timely removal to achieve source control. We report a case of tooth aspiration successfully retrieved with a cryoprobe.

## Graphical abstract

A 66‐year‐old man presented with sepsis secondary to post‐obstructive pneumonia following aspiration of a canine tooth. The tooth was successfully removed with a cryoprobe.

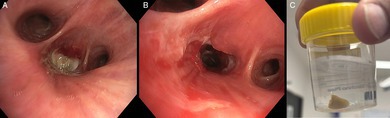

A 66‐year‐old man presented to hospital with a 1‐week history of a productive cough following inadvertent aspiration of a canine tooth while eating a crumpet. On examination, he was febrile (38.2°C), tachycardic (heart rate 125 beats per minute), hypotensive (systolic blood pressure 90 mmHg), with coarse crepitations audible at the right lung base. His white cell count was 10.6 × 10^9^/L and his CRP was 308 mg/L. Chest x‐ray (Figure 1) and subsequent computed tomography (Figure 2) demonstrated a lucency at the orifice of the right lower lobe bronchus, with post‐obstructive consolidation and collapse. He was treated with intravenous antibiotics and underwent a flexible bronchoscopy which demonstrated a tooth lodged within the right lower lobe bronchus resulting in distal obstruction. A 1.1 mm cryoprobe was used to remove the tooth, and extensive mucopurulent secretions were suctioned (Figure 3).

**FIGURE 1:**
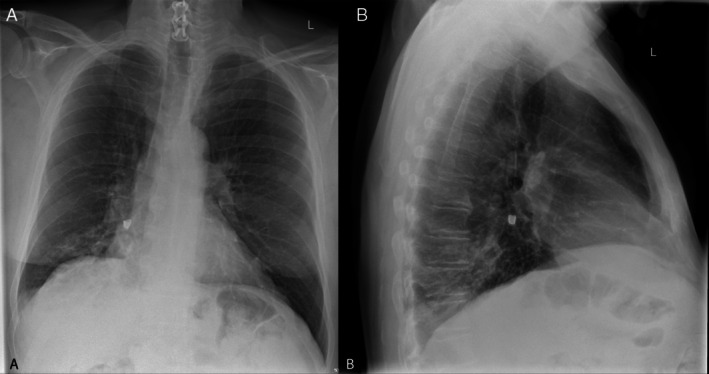
Anteroposterior (A) and lateral chest x‐ray (B) demonstrating a lucency projecting over the right lower lobe with evidence of distal pulmonary infiltrate and lower lobe collapse.

**FIGURE 2:**
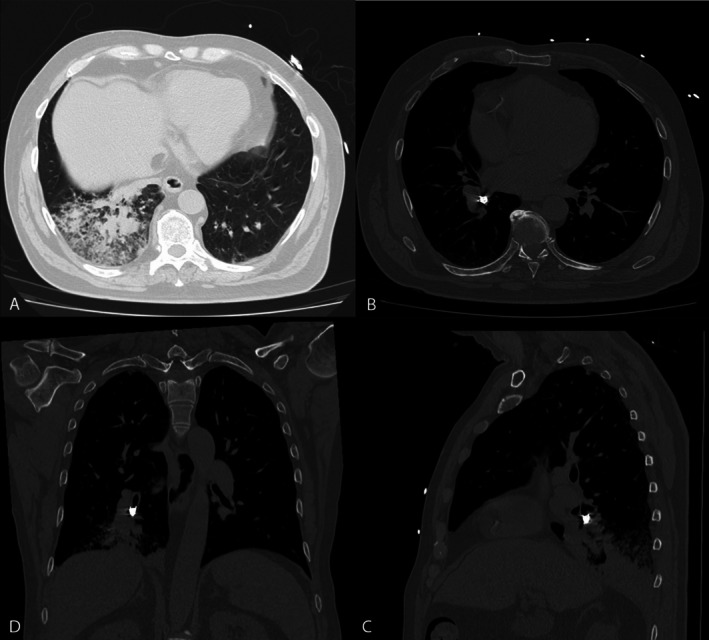
(A–D clockwise from top left): Computed tomography of the chest demonstrating a lucency within the right lower lobe bronchus with associated distal consolidation and partial right lower lobe collapse. Metallic artefact secondary to a dental crown is noted.

**FIGURE 3:**
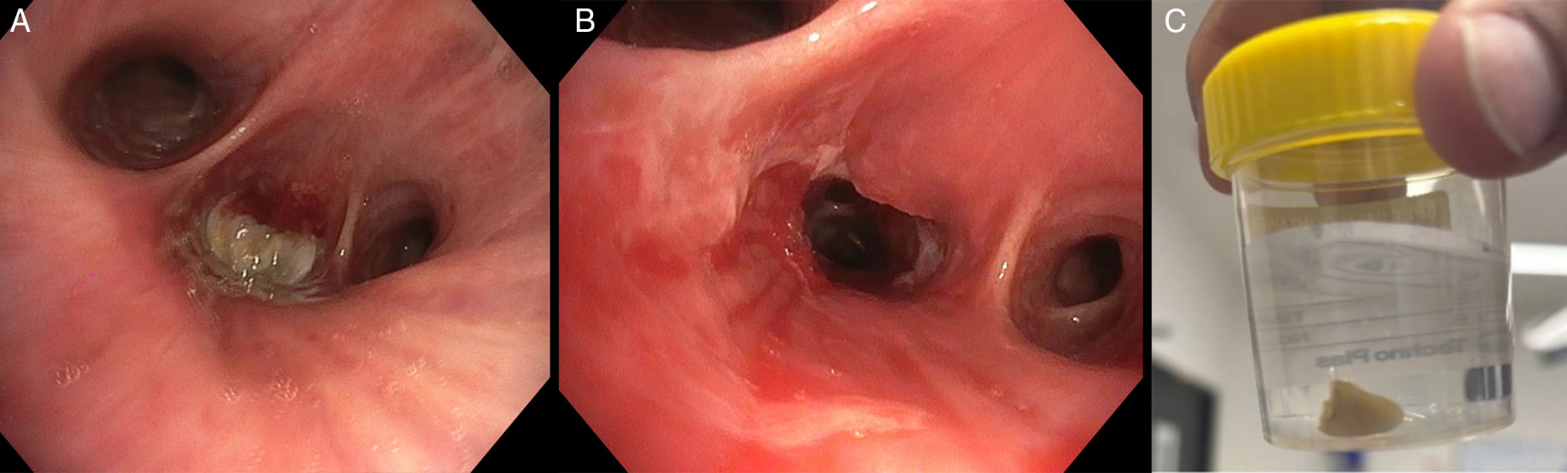
Bronchoscopic images of a tooth obstructing the right lower lobe bronchus (A), the right lower lobe bronchus once cleared of endobronchial obstruction (B) and the tooth post extraction (C).

Foreign body aspiration is relatively uncommon in adults. Removal of the aspirated material is essential to prevent complications including airway mucosal ulceration and post‐obstructive pneumonia. While at times either rigid bronchoscopy or traditional grasping devices (forceps, basket) may be suitable to remove aspirated teeth, a cryoprobe can be an effective alternative if the tooth pulp is exposed or there is mucus adherent to it [1, 2].

## Author Contributions

Matthew Donnan, Melanie Wong and Elina Chi: conceptualisation, draft writing, review, editing. Dominic Keating: supervision, writing – review and editing.

## Consent

The authors declare that written informed consent was obtained for the publication of this manuscript and accompanying images using the consent form provided by the Journal.

## Conflicts of Interest

The authors declare no conflicts of interest.

## Data Availability

Data sharing not applicable to this article as no datasets were generated or analysed during the current study.

## References

[1] H. Ishimoto , N. Sakamoto , S. Moriyama , et al., “Removal of an Aspirated Tooth From the Bronchus Using a Cryoprobe: A Case Report,” Respirology Case Reports 9, no. 12 (2021): e0880. 34853696 10.1002/rcr2.880 PMC8612864

[2] H. Azam and P. Wu , “Bronchoscopic Retrieval of an Aspirated Tooth Following High‐Speed Motor Vehicle Accident,” Respirology Case Reports 12, no. 8 (2024): e01444. 39086723 10.1002/rcr2.1444 PMC11290951

